# Development of an initial training and evaluation programme for manual lower limb muscle MRI segmentation

**DOI:** 10.1186/s41747-024-00475-9

**Published:** 2024-07-25

**Authors:** Jasper M. Morrow, Sachit Shah, Lara Cristiano, Matthew R. B. Evans, Carolynne M. Doherty, Talal Alnaemi, Abeer Saab, Ahmed Emira, Uros Klickovic, Ahmed Hammam, Afnan Altuwaijri, Stephen Wastling, Mary M. Reilly, Michael G. Hanna, Tarek A. Yousry, John S. Thornton

**Affiliations:** 1 Department of Neuromuscular Diseases, Queen Square UCL Institute of Neurology, London, UK; 2 Queen Square Centre for Neuromuscular Diseases, National Hospital for Neurology and Neurosurgery, UCLH, London, WC1N 3BG UK; 3 Lysholm Department of Neuroradiology, The National Hospital for Neurology and Neurosurgery, UCLH, London, UK; 4 Neuroradiological Academic Unit, Queen Square UCL Institute of Neurology, London, UK; 5 Department of Radiology and Pediatric Neurology, Policlinico Universitario A. Gemelli, Università Cattolica del Sacro Cuore, Rome, Italy; 6 Department of Biomedical Imaging and Image-Guided Therapy, Medical University of Vienna, Vienna, Austria

**Keywords:** Benchmarking, Magnetic resonance imaging, Muscle (skeletal), Neuromuscular diseases, Thigh

## Abstract

**Background:**

Magnetic resonance imaging (MRI) quantification of intramuscular fat accumulation is a responsive biomarker in neuromuscular diseases. Despite emergence of automated methods, manual muscle segmentation remains an essential foundation. We aimed to develop a training programme for new observers to demonstrate competence in lower limb muscle segmentation and establish reliability benchmarks for future human observers and machine learning segmentation packages.

**Methods:**

The learning phase of the training programme comprised a training manual, direct instruction, and eight lower limb MRI scans with reference standard large and small regions of interest (ROIs). The assessment phase used test–retest scans from two patients and two healthy controls. Interscan and interobserver reliability metrics were calculated to identify underperforming outliers and to determine competency benchmarks.

**Results:**

Three experienced observers undertook the assessment phase, whilst eight new observers completed the full training programme. Two of the new observers were identified as underperforming outliers, relating to variation in size or consistency of segmentations; six had interscan and interobserver reliability equivalent to those of experienced observers. The calculated benchmark for the Sørensen-Dice similarity coefficient between observers was greater than 0.87 and 0.92 for individual thigh and calf muscles, respectively. Interscan and interobserver reliability were significantly higher for large than small ROIs (all *p* < 0.001).

**Conclusions:**

We developed, implemented, and analysed the first formal training programme for manual lower limb muscle segmentation. Large ROI showed superior reliability to small ROI for fat fraction assessment.

**Relevance statement:**

Observers competent in lower limb muscle segmentation are critical to application of quantitative muscle MRI biomarkers in neuromuscular diseases. This study has established competency benchmarks for future human observers or automated segmentation methods.

**Key points:**

• Observers competent in muscle segmentation are critical for quantitative muscle MRI biomarkers.

• A training programme for muscle segmentation was undertaken by eight new observers.

• We established competency benchmarks for future human observers or automated segmentation methods.

**Graphical Abstract:**

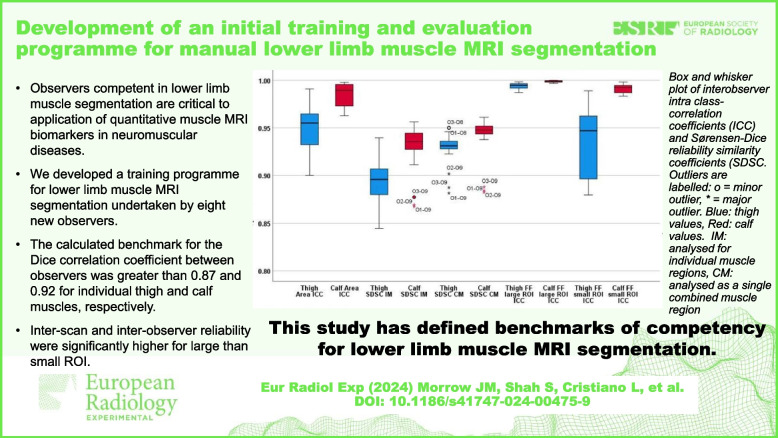

## Background

In the field of neuromuscular diseases where chronic disease is associated with a loss of muscle volume and fat replacement, increasing attention has been recently given to quantitative assessment of intramuscular fat [1–7]. New experimental therapies have recently become available for the treatment of many neuromuscular diseases [8–10] necessitating reliable clinical trial outcome measures with attestable longitudinal responsiveness.

Quantitative magnetic resonance imaging (MRI) has been shown to be a sensitive noninvasive measure of muscle pathology and disease progression in neuromuscular diseases, providing information on muscle structure and physiopathology [1–7]. Despite new developments in automation, analysis usually relies fundamentally on a manual segmentation of regions of interest (ROIs) to extract anatomic specific information. Manual muscle segmentation requires accurate offline analyses of, often, axial images by means of contour tracing of each individual muscle of interest, drawing close to the lines of the muscle aponeurosis and fascia without including the intermuscular and subcutaneous adipose tissue [11]. High interscan reliability is a crucial component of outcome measure responsiveness and depends upon reproducibility of both the image acquisition and analysis. The expertise of the observer performing manual segmentations is clearly therefore important, but consistency can be challenging since subjective choices with regards to boundary definitions are involved, particularly so in data sets exhibiting marked muscle-fat replacement. However, neither a training programme, nor a segmentation performance evaluation framework has previously been systematically assessed. Furthermore, in large clinical trials, time consuming manual segmentation can be a significant bottle neck to data analysis, which could be mitigated by utilising multiple observers, provided their segmentations are sufficiently consistent. It is therefore necessary to investigate systematically both interscan (with the same observer) and interobserver reliability.

In addition to direct analyses of MRI data to produce outcome measures, manual region labelling is a core requirement in generating training and test data for the development of automated analysis tools using machine learning approaches such as deep learning with convolutional neural networks [12]. These methods require large libraries of expertly labelled training image data sets, and the consistency of these labels may impact significantly on their performance. The scheme we describe herein may both assist in this regard and provide a framework for systematically evaluating machine segmentation implementations against the best-case performance of expert human observers.

So far, only a few studies have investigated inter or intraobserver reliability of muscle fat quantification using quantitative MRI [6, 13, 14]. These studies have provided important information but were mainly focused on the assessment of one single muscle, or one level, either distal or proximal. One other study evaluated intraobserver and interobserver variability of manual segmentation for the assessment of muscle fat content at the level of L3 vertebra (used as a landmark) in a general population-based sample [15]. Although we have previously evaluated scan-rescan over 2 weeks and interobserver reliability between two observers of skeletal muscle fat-fraction using quantitative MRI measures in 18 healthy subjects and found these to be highly reliable [16], to our knowledge, this has never been systematically assessed in both healthy and pathological muscles at thigh and calf level. Whilst this reliability analysis was performed using small ROI, a larger ROI encompassing the whole muscle cross section proved more responsive over 12 months in patients with Charcot Marie Tooth disease and inclusion body myositis [17], but the relative reliability of small and large ROI has not been examined. In 1998, a seminal paper [18] evaluated the reproducibility of quantitative MRI measurements for monitoring the treatment of multiple sclerosis and highlighted that for both manual segmentation and semiautomated segmentation techniques, comprehensive operator training improves the reproducibility of the analyses and should be considered as a standard procedure for clinical trials in which individual lesions are delineated manually or semiautomatically.

In the present study, we aimed to develop a training programme such that new observers were adequately trained and could demonstrate competence prior to commencing manual muscle segmentation of new datasets including healthy volunteers and patients, at thigh and calf level, to improve longitudinal reliability. Specifically, we aimed to establish a scheme to identify newly trained observers who were underperforming in terms of reliability metrics, identify those performing at the same level as experienced observers and establish reliability benchmarks for future observers and machine learning segmentation packages.

## Methods

### Study design

**Fig. 1:**
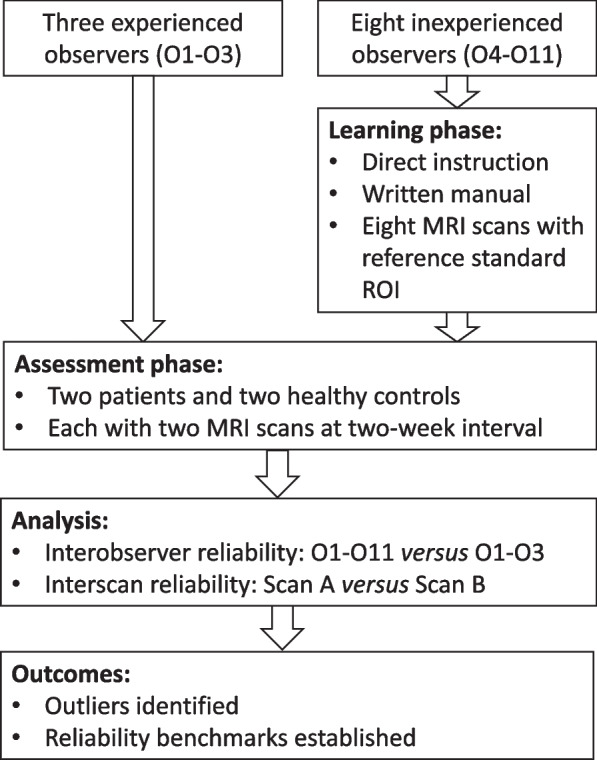
Study flow chart. *MRI* Magnetic resonance imaging, *ROI* Region of interest

A systematic lower limb muscle segmentation training programme was designed prospectively in two parts: a learning phase and an assessment phase. The study design is summarised in Fig. 1. Both phases utilised three-point Dixon acquisitions at thigh level and calf level from previously published studies [16, 17] with ethics committee approval (Joint UCL/UCLH Committees on the Ethics of Human Research, reference: 08/H0715/117) and written informed consent from all participants with inclusion and exclusion criteria as described previously [16, 17]. As in these studies, muscle segmentation was performed using ITK-SNAP [19] freely available segmentation software commonly used in a research setting.

### MRI methods

Segmentation was performed on a single prespecified slice of a two-dimensional gradient-echo image (echo time 3.45 ms, repetition time 100 ms, flip angle 10°, bandwidth 420 Hz/pixel, number of excitations 4, 10 × 10-mm slices with 10-mm gap) acquired as part of a three-point Dixon acquisition on a 3-T scanner (TIM Trio, Siemens, Erlangen, Germany). Separate acquisitions at mid-thigh and mid-calf level were used with axial-slice matrices and fields of view 512 × 256 and 400 × 200 mm^2^ for thigh-level images and 512 × 240 and 400 × 188 mm^2^ for calf-level images. The segmentations were transferred to the inherently coregistered fat fraction maps [16], and the mean fat fraction value and cross-sectional area for each segmentation was automatically extracted for analysis.

### Learning phase

The learning phase of the programme comprised access to a written instruction manual, direct instruction from a tutor (S.S.) experienced in muscle segmentation, and access to the learning set of acquisitions including reference standard ROIs. The instruction manual was written by two experienced observers (J.M., S.S.) and contained information on thigh and calf muscle anatomy with links to further resources, instructions on ITK-SNAP software, detailed explanations of small and large ROI segmentations with model examples, and specific notes with regards to individual muscles. This was complemented by direct instruction from the tutor which comprised an initial session to review the training programme, software and muscle anatomy according to the needs of the learner, supervision and input as needed throughout the learning phase, and a final review of practice segmentations to confirm when the new observer was ready to undertake the final assessment.

The learning set of image data comprised eight previously acquired MRI examinations of the lower limb muscles including single time-point data from four patients: two with Charcot-Marie-Tooth disease type 1A and two with inclusion body myositis [17] and acquisitions from two healthy controls who had undergone two separate MRI examinations performed at a two-week interval [16]. The new observers used this dataset to define ROIs on each of the eight examinations, on prespecified slices at both thigh and calf level. For each of the eight MRI examinations, the trainees were provided with ‘reference standard’ segmentations defined by two experienced observers (J.M., S.S.) by consensus according to the approach outlined in the instruction manual. Specifically, individual muscles were segmented bilaterally on the pre-specified slice at thigh and calf levels (rectus femoris, vastus lateralis, vastus intermedius, vastus medialis, semimembranosus, semitendinosus, biceps femoris, adductor magnus, sartorius, gracilis, tibialis anterior, peroneus longus, lateral gastrocnemius, medial gastrocnemius, soleus, and tibialis posterior muscles). ‘Large’ and ‘small’ ROI segmentations were performed. The large segmentations encompassed the whole muscle cross section, excluding the voxels at the muscle boundary, whilst the small segmentations were a single 12-voxel diameter circular ROI placed within muscle belly to ensure no contamination with fascia, fat, or blood vessels (for example, see [17]).

### Assessment phase

The assessment phase was based on eight further MRI examinations of the lower limb muscles. These comprised two separate MRI examinations performed at a 2-week interval from four subjects: two patients with inclusion body myositis and two healthy controls. This allowed assessment of interscan reliability for each observer as well as inter-observer reliability for the same acquisitions. For this formal assessment, observers independently performed muscle segmentations on these eight MRI examinations on the right limb only, with the left lower limb reserved in case a learner needed to repeat the assessment. Observers were instructed to compare the two examinations for each subject to ensure consistency of segmentation. Observers were instructed to define the ROI at thigh and calf level, on pre-specified slices selected by an experienced observer (J.M.) to be the same distance from the knee joint for both examinations.

### Implementation of the training programme

Three experienced observers (O1−O3) independently undertook the assessment phase of the training programme according to the methods described in the instruction manual. Eight medical doctors inexperienced in muscle segmentation undertook the training programme, referred to as newly trained observers (O4−O11), including six training in radiology and two training in neurology. After the newly trained observers completed the assessment phase, their ROIs were checked by J.M. for ROI labelling errors, and these were corrected, prior to automated extraction of muscle fat fraction and size metrics.

### Data analysis

**Fig. 2:**
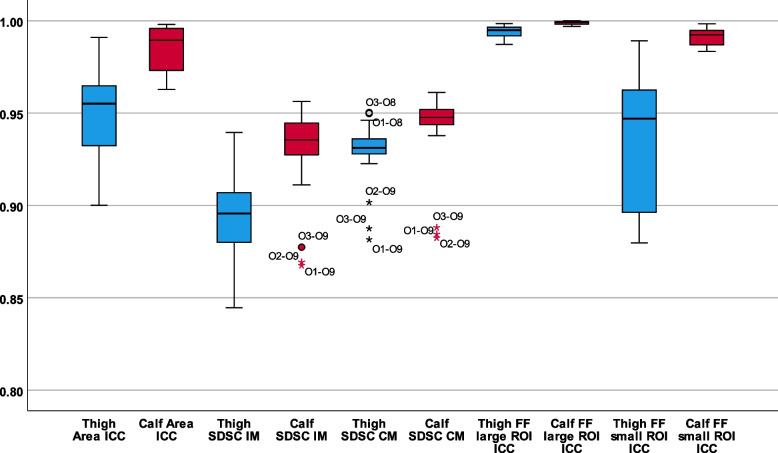
Box and whisker plots of interobserver ICC and SDSC. o = minor outlier; * = major outlier. Thigh values in blue, calf values in red. *CM* Combined muscle region, *FF* Fat fraction, *ICC* Intraclass correlation coefficient, *IM* Individual muscle regions, *ROI* Region of interest, *SDSC* Sørensen-Dice similarity coefficient

Statistical analysis was performed using SPSS 26 (IBM, Armonk, NY, USA). To assess the consistency of segmentation between observers, each observer (O1−O11) was compared pairwise with the experienced observers (O1−O3) (Fig. 2, Table 1), by the following interobserver metrics across the eight scans at thigh and calf level separately, totalling 80 thigh muscle and 48 calf muscle ROI per observer:

- Comparison of the mean value for large ROI size, large ROI fat fraction, and small ROI fat fraction;
- Intraclass correlation coefficient (ICC) for large ROI size, large ROI fat fraction, and small ROI fat fraction;
- Sørensen-Dice similarity coefficient (SDSC) for large ROI, both for individual muscles as segmented, and if all muscles combined into a single segmentation (see below).

**Table 1:** Interobserver reliability assessed by ICC and SDSC for thigh and calf muscles

Pair	Thigh ICC			Thigh SDSC		Calf ICC			Calf SDSC	
	ROI size	Large FF	Small FF	IM	CM	ROI size	Large FF	Small FF	IM	CM
O1 *versus* O2	0.963	0.995	0.894	0.901	0.932	0.980	0.998	0.998	0.932	0.944
O1 *versus* O3	0.974	0.997	0.964	0.922	0.941	0.995	1.000	0.993	0.946	0.951
O2 *versus* O3	0.964	0.998	0.915	0.905	0.935	0.971	0.998	0.993	0.941	0.952
O4 *versus* O1	0.923	0.987	0.971	0.876	0.934	0.989	0.999	0.984	0.925	0.943
O4 *versus* O2	0.962	0.992	0.908	0.874	0.923	0.968	0.997	0.986	0.911	0.940
O4 *versus* O3	0.936	0.991	0.954	0.877	0.928	0.993	0.999	0.984	0.927	0.945
O5 *versus* O1	0.955	0.994	0.978	0.898	0.933	0.995	1.000	0.995	0.936	0.944
O5 *versus* O2	0.946	0.992	0.891	0.900	0.935	0.973	1.000	0.997	0.935	0.949
O5 *versus* O3	0.955	0.994	0.936	0.900	0.930	0.997	0.998	0.992	0.943	0.952
O6 *versus* O1	0.912	0.988	0.961	0.892	0.931	0.995	1.000	0.988	0.939	0.947
O6 *versus* O2	0.955	0.988	0.896	0.890	0.926	0.973	0.999	0.992	0.930	0.947
O6 *versus* O3	0.900	0.989	0.936	0.883	0.928	0.997	0.998	0.983	0.943	0.952
O7 *versus* O1	0.956	0.995	0.973	0.920	0.946	0.997	1.000	0.986	0.946	0.951
O7 *versus* O2	0.928	0.992	0.896	0.888	0.928	0.975	0.999	0.988	0.933	0.948
O7 *versus* O3	0.935	0.993	0.950	0.894	0.930	0.997	0.998	0.985	0.949	0.953
O8 *versus* O1	0.991	0.998	0.986	0.940	0.950	0.995	1.000	0.995	0.952	0.956
O8 *versus* O2	0.967	0.995	0.883	0.909	0.937	0.970	1.000	0.997	0.936	0.948
O8 *versus* O3	0.981	0.997	0.950	0.934	0.950	0.998	0.998	0.993	0.956	0.961
O9 *versus* O1	0.937	0.994	0.989	0.845	**0.882**	0.989	1.000	0.992	**0.869**	**0.882**
O9 *versus* O2	0.966	0.996	0.891	0.873	**0.902**	0.971	0.999	0.993	**0.868**	**0.884**
O9 *versus* O3	0.930	0.995	0.947	0.845	**0.888**	0.988	0.998	0.996	**0.877**	**0.888**
O10 *versus* O1	0.985	0.997	0.988	0.920	0.938	0.997	0.999	0.996	0.946	0.953
O10 *versus* O2	0.979	0.997	0.880	0.916	0.938	0.972	1.000	0.995	0.934	0.950
O10 *versus* O3	0.960	0.997	0.950	0.904	0.931	0.997	0.998	0.993	0.950	0.955
O11 *versus* O1	0.914	0.992	0.948	0.875	0.927	0.989	1.000	0.988	0.928	0.938
O11 *versus* O2	0.957	0.996	0.891	0.896	0.935	0.963	0.999	0.985	0.922	0.938
O11 *versus* O3	0.923	0.995	0.924	0.885	0.930	0.992	0.997	0.992	0.936	0.944
Mean	0.950	0.994	0.935	0.895	0.929	0.986	0.999	0.991	0.930	0.941
*p*-TvC	< 0.001	< 0.001	< 0.001	< 0.001	0.014					

The first three rows are between experienced observers. Underperforming outliers are in bold. *CM* Analysis for a combined muscle region, *FF* Fat fraction, *ICC* Intraclass correlation coefficient, *IM* Analysis for individual muscle regions, *p-TvC* *p*-value comparing thigh and calf values of reliability, *ROI* Region of interest, *SDSC* Sørensen-Dice similarity coefficient

The SDSC provides a measure of the similarity of two sets, in this case voxels included within a region of interest, and is given by the formula:

$$\frac{2 |\mathrm{A }\cap \mathrm{ B}|}{|{\text{A}}|+|{\text{B}}|}$$

The result is a value between 0 and 1 where 0 indicates no voxels in common between the two segmentations and 1 indicates identical voxels in each segmentation. The mean SDSC coefficient was calculated both for the individual muscles as segmented (SDSC individual muscle), but also by considering all muscles as a single segmentation, referred to as ‘SDSC combined muscle’. As for the other interobserver reliability metrics, this was assessed pairwise between each observer (O1−O11) and experienced observers (O1−O3), with mean values across the 8 scans presented separately for thigh and calf levels.

In addition, to assess the internal consistency of each observer’s segmentation of the four test–retest scans in the assessment phase, interscan reliability metrics were calculated for thigh and calf level muscles separately across 40 thigh muscle (4 subjects × 10 muscles per subject) and 24 calf muscle (4 subjects × 6 muscles per subject) ROIs. ICCs were calculated for large ROI size (effectively muscle cross-sectional area), large ROI fat fraction, and small ROI fat fraction. Bland–Altman analysis [20] was performed by calculating mean value, mean interscan difference and mean interscan standard deviation for large ROI size, large ROI fat fraction, and small ROI fat fraction. These were calculated independently for each observer (O1−O11).

Observers were assessed by identifying observers who were outliers with respect to any of the inter-observer and interscan reliability metrics through analysis of box and whisker plots for all observers. An outlier was defined as falling more than 1.5 × interquartile ranges outside the first or third quartiles: a major outlier if greater than 3 × interquartile ranges, minor if between 1.5 × and 3 × interquartile ranges. Underperforming outliers were those where the performance was in the direction of lower reliability (*e.g.*, lower ICC or higher interscan standard deviation than other observers). By considering the above, newly trained observers were assessed as being competent to perform muscle segmentation or needing further training prior to repeat assessment on the contralateral limb. Finally, benchmark levels of reliability were determined to assess future learners undertaking this training programme, calculated as the lower bound of the 95% confidence interval of values observed for each reliability measurement (mean -1.96 standard deviation).

In addition to assessing observer performance, both interobserver and interscan reliability metrics were analysed to determine relative reliability of thigh *versus* calf muscle segmentation, small ROI *versus* large ROIs, and patient scans *versus* control scans. Normal data distribution was assessed using Shapiro–Wilk test. Significant differences were assessed with a 2-tailed Student *t*-test; *p*-values lower than 0.050 were considered significant.

## Results

### Training programme

Three experienced observers first undertook the assessment phase independently according to the method detailed in the instruction manual. Subsequently eight previously inexperienced observers completed the training programme including the learning phase and the assessment phase. The average time in the learning phase was 30 h distributed over 1−4 weeks.

### Interobserver reliability

All observers (O1−O11) were compared with the three experienced observers (O1−O3). ICCs and SDSCs are given in Table 1 and as a box and whisker plot in Fig. 2. Reliability was greater for calf muscle segmentation than thigh muscle segmentations (*p* from 0.014 to < 0.001).

### Interscan reliability

Using the ROIs defined by each observer upon the scan-rescan data sets from the four subjects in the assessment phase, inter-scan reliability for large ROI size, large ROI fat fraction and small ROI fat fraction is given for both experienced observers (O1−O3) and newly trained observers (O4−O11) in thigh muscles (Table 2) and calf muscles (Table 3). The values presented are with each of the individual muscle ROIs as the data points (*n* = 40 for thigh;* n* = 24 for calf).

**Table 2:** Interscan reliability in thigh muscles (*n* = 40 for all)

	ROI size (voxels)				Large ROI fat fraction (% ff)				Small ROI fat fraction (% ff)			
	BL mean	IS Diff	IS s.d	ICC	BL mean	IS Diff	IS s.d	ICC	BL mean	IS Diff	IS s.d	ICC
O1	1,488	-0.3%	12.5%	0.981	21.1	-0.70	2.05	0.997	18.7	-0.03	6.5	0.968
O2	1,411	-2.8%	12.8%	0.980	20.2	-0.69	2.30	0.996	17.6	-0.22	9.2	0.937
O3	1,449	**-3.3%**	12.5%	0.981	20.6	-0.51	1.92	0.997	19.3	0.07	3.2	0.992
O4	**1,576**	**1.7%**	**20.8%**	**0.949**	21.9	0.12	**4.25**	**0.985**	**20.0**	**1.35**	9.0	0.947
O5	1,428	-0.8%	13.2%	0.980	20.6	-0.22	2.52	0.995	18.8	**1.47**	9.5	0.940
O6	1,488	-0.5%	18.1%	0.962	21.7	-0.16	2.68	0.994	19.1	**-2.11**	10.0	0.934
O7	1,479	-0.5%	11.5%	0.984	21.1	-0.25	2.25	0.996	18.8	-0.02	2.8	0.995
O8	1,477	-0.8%	12.1%	0.983	20.5	-0.58	1.80	0.997	18.7	0.35	9.4	0.933
O9	1,442	-1.8%	19.8%	0.955	20.6	-0.70	2.64	0.995	19.0	**-0.98**	9.1	0.939
O10	1,436	-0.9%	11.4%	0.985	20.7	-0.54	1.89	0.997	19.1	0.33	8.0	0.953
O11	1,424	-1.4%	9.5%	0.990	20.3	-0.06	2.05	0.997	19.4	0.07	4.7	0.985
Mean	1,463	-1.0%	14.0%	0.975	20.9	-0.36	2.40	0.995	19.0	0.03	7.4	0.957

Identified outliers are in bold. *BL* Baseline, *ICC* Intraclass correlation coefficient, *IS Diff* Mean interscan difference, *IS s.d.* Interscan standard deviation, *ROI* Region of interest

**Table 3:** Interscan reliability in calf muscles (*n* = 24 for all)

	ROI size (voxels)				Large ROI fat fraction (% ff)				Small ROI fat fraction (% ff)			
	Mean	IS Diff	IS s.d	ICC	Mean	IS Diff	IS s.d	ICC	Mean	IS Diff	IS s.d	ICC
O1	1,648	-0.8%	11.3%	0.993	13.6	-0.41	1.46	0.998	12.8	-1.06	2.1	0.997
O2	1,617	0.8%	8.7%	0.994	13.9	-0.57	1.16	0.999	12.6	-0.29	1.6	0.998
O3	1,638	1.2%	10.0%	0.993	13.5	-0.30	1.18	0.999	13.3	0.16	5.0	0.985
O4	**1,753**	2.0%	11.6%	**0.987**	13.6	**0.33**	1.12	0.999	12.7	-0.58	1.6	0.999
O5	1,629	2.3%	8.7%	0.997	13.6	-0.44	1.30	0.999	12.8	-0.08	1.8	0.998
O6	1,665	3.0%	11.5%	0.994	13.6	-0.23	1.35	0.999	**11.7**	0.18	3.4	0.995
O7	1,694	0.5%	8.6%	0.996	13.7	-0.29	0.95	0.999	12.8	-1.12	2.6	0.997
O8	1,662	1.4%	7.2%	0.996	13.5	-0.31	1.13	0.999	12.9	-0.14	1.8	0.998
O9	**1,341**	-1.2%	14.9%	**0.985**	13.5	-0.42	0.97	1.000	13.0	0.81	3.3	0.992
O10	1,652	2.0%	7.2%	0.997	13.5	-0.38	0.94	0.999	13.5	-1.17	3.7	0.993
O11	1,635	1.2%	8.9%	0.992	13.7	-0.16	1.34	0.999	13.7	-1.42	4.3	0.990
Mean	1,630	1.1%	9.9%	0.993	13.6	-0.29	1.17	0.999	12.9	-0.43	2.8	0.995
*p*-TvC	NA	< 0.001	0.005	< 0.001	NA	0.562	< 0.001	0.001	NA	0.234	< 0.001	< 0.001

Identified outliers are in bold. *BL* Baseline, *ICC* Intraclass correlation coefficient, *IS Diff* Mean interscan difference, *IS s.d.* Interscan standard deviation, *p-TvC* *p*-value comparing thigh and calf values of reliability, *ROI* Region of interest

### Observers being underperforming outliers

None of the experienced observers (O1−O3) were identified as underperforming outliers with respect to any of the interobserver and interscan reliability metrics. The value of the interscan difference for thigh muscle area was -3.3% for observer O3 was identified as a minor outlier, even though this difference was not statistically significant (*p* = 0.101) and both interscan standard deviation and ICC for this measure were comparable to the other experienced observers and better than the overall mean for these values.

**Fig. 3:**
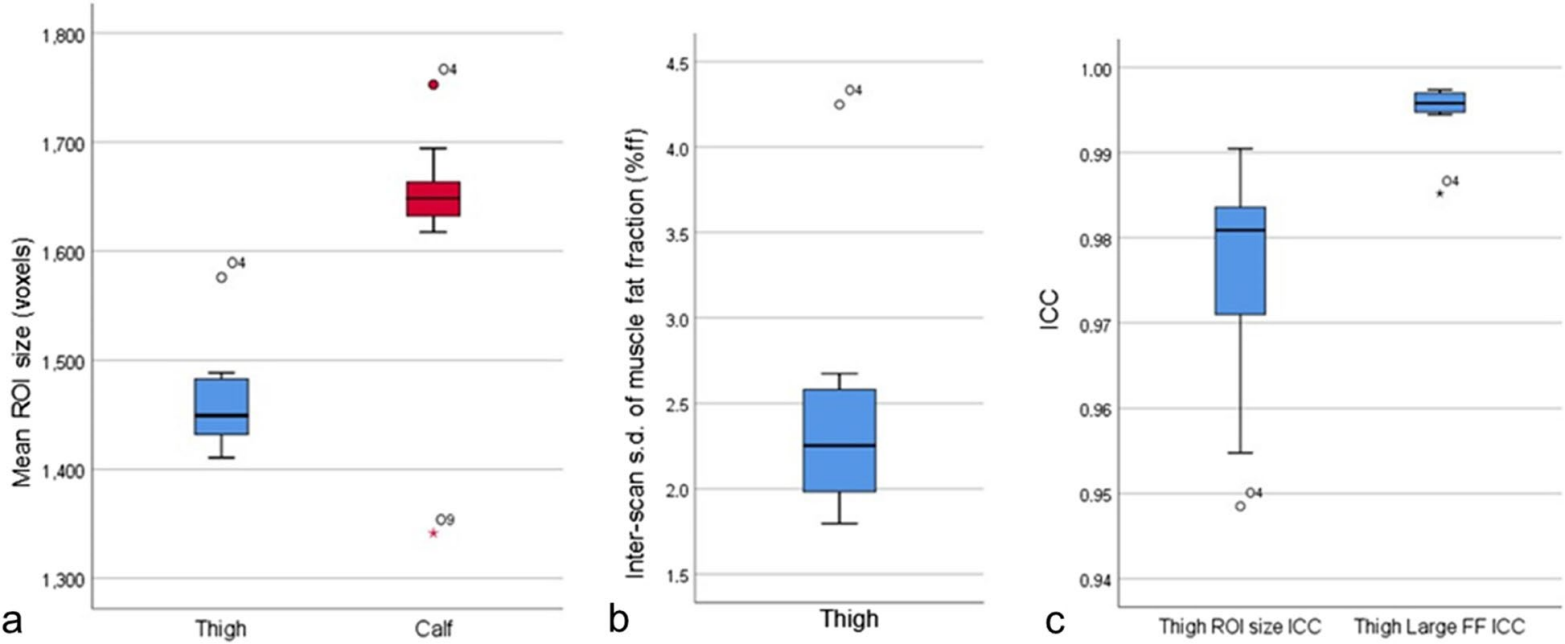
Results highlighting O4 as an underperforming outlier. **a** Box-whisker plot of mean large-ROI size for baseline scans identified O4 as a minor outlier with large-ROI for both thigh and calf muscles, whilst O9 was a major outlier with small calf muscle ROI size. **b** Box-whisker plot of values for all observers of interscan standard deviation of large-ROI fat-fraction at thigh level. O4 is a minor outlier for this metric demonstrating lower interscan reliability. **c** Interscan reliability of thigh large ROI assessed by ICC of large-ROI size (cross-sectional area) and fat fraction. O4 is identified as a minor outlier for ROI size ICC and major outlier for large-ROI FF ICC. o = minor outlier; * = major outlier. *ROI *Region of interest, *FF* Fat fraction, *ICC* Intraclass correlation coefficient

**Fig. 4:**
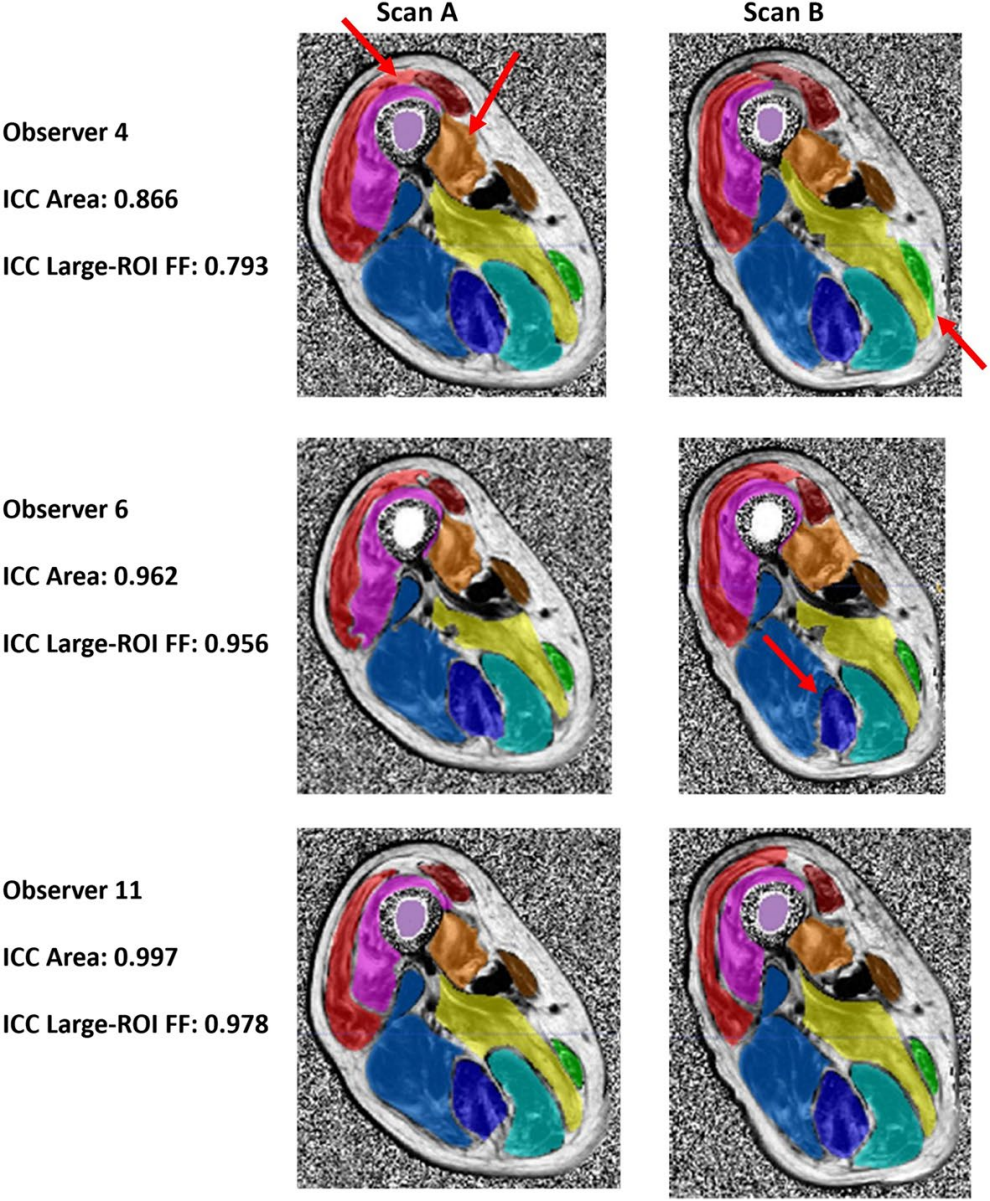
Examples of new observers’ thigh-muscle test–retest large ROIs in an inclusion body myositis patient. Areas where ROI definitions differ between scans are indicated by red arrows. Observer 4 had the lowest level of agreement, observer 6 had a typical level of agreement, whilst observer 11 had the best level of agreement for this pair of scans. *FF* Fat fraction, *ICC* Intraclass correlation coefficient, *ROI* Region of interest

Two of the newly trained observers were identified as significant outliers. O4 segmented significantly larger ROI than other observers (Fig. 3a) for both thigh muscles (1,576 *versus* 1,452 voxels, *p* < 0.001) and calf muscles (1,753 *versus* 1,618 voxels, *p* < 0.001). On reliability assessments, the large thigh ROI for O4 were also outliers for interscan thigh standard deviation (4.3% *versus* 2.4%, Fig. 3b) and lowest ICC (Fig. 3c). Visual review of thigh ROI demonstrated interscan variation in muscle boundaries (arrows in Fig. 4). The large calf muscle segmentations for O4 were an outlier for interscan reliability of ROI size (Table 3), but the derived large ROI fat fraction showed an excellent interscan reliability (ICC 0.999).

**Fig. 5:**
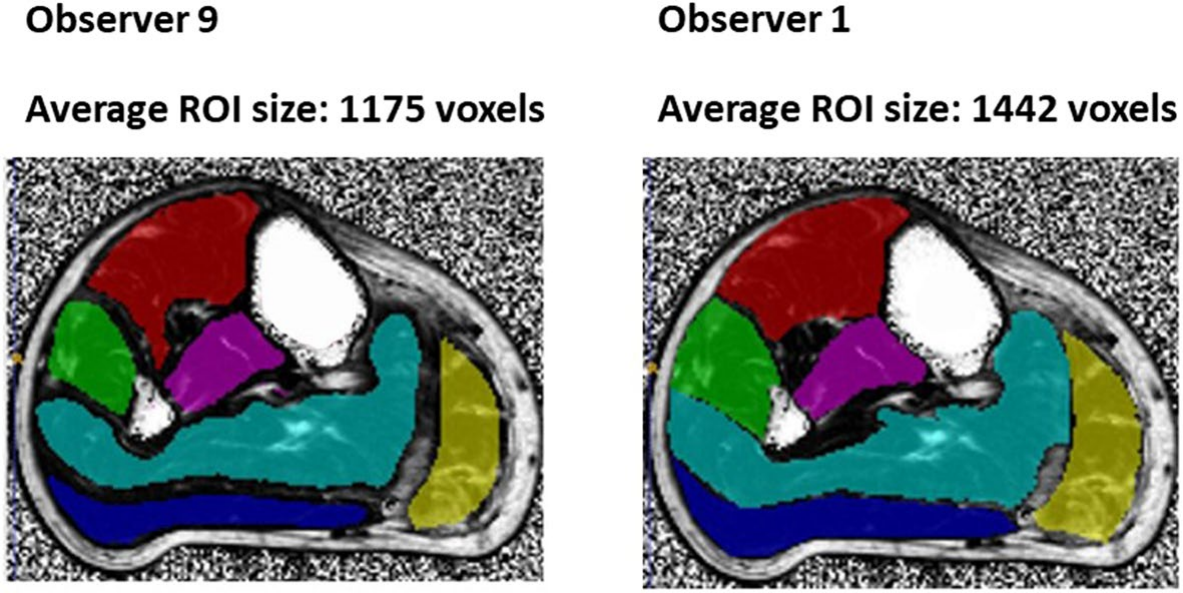
Large ROI in calf muscles of example new observer and experienced observer. Observer 9 defined significantly smaller ROIs (regions of interest) for calf muscles than the experienced observers for all scans (all *p* ≤ 0.001), resulting in low Sørensen-Dice coefficients with respect to those observers (all less than 0.90)

The second newly trained observer identified as a significant outlier was O9: the calf muscle segmentations were significantly smaller than those of other observers at 1,341 *versus* 1,659 voxels (*p* < 0.001; Fig. 3a, Fig. 5). This was associated with a reduced interscan reliability for ROI size (O9, ICC 0.985; mean of O1−O11, ICC 0.993; Table 3). However, interscan reliability of large ROI calf fat fraction for this observer was excellent (ICC 1.000). In terms of interobserver reliability, the SDSCs against the three experienced observers were all major outliers (Fig. 1, bold values in Table 1), whether the muscles were considered individually or combined. For O9’s large thigh muscle ROI, the interscan ICC for area was also a minor outlier at 0.985 (Fig. 3a), and interobserver SDSCs against the experienced observers were also lowest for individual muscles and were minor outliers compared with the other observers for combined muscles.

Based on both the qualitative and quantitative assessment, O4 and O9 were considered to have not reached the necessary standard of reliability and competence to pass the segmentation training programme. All the other newly trained observers (O5−8, O10−11) had reliability metrics indistinguishable from experienced observers (O1−O3) and were therefore passed as competent at performing muscle segmentation. O4 and O9 received feedback on their performance and subsequently successfully met the required standard and were assessed as competent.

### Relative reliability of thigh *versus* calf muscle segmentation and small *versus* large ROIs

Both interscan and interobserver reliability were higher for calf muscle metrics than thigh muscle metrics (Tables 1, 2, and 3 Fig. 2, all *p* < 0.001). For example, the mean interscan ICC for ROI size was 0.975 for thigh muscles, 0.993 for calf muscles, whilst interobserver ICC for ROI size was 0.950 for thigh muscles, 0.986 for calf muscles. SDSCs were similarly significantly higher for calf muscles than thigh muscles (Table 1).

Fat fraction measurements obtained from large ROIs encompassing the whole muscle cross section had greater interscan and interobserver reliability than values obtained using small ROIs (all *p* < 0.001; Tables 1, 2, and 3; Fig. 2). For this reason, for the assessment of newly trained observers as competent and setting reliability benchmarks for competency, only results obtained from large ROI were considered.

### Proposed benchmark values of interobserver and intraobserver reliability metrics

Excluding the results of O4 and O9, reliability metrics from the remaining nine observers were assessed to determine the proposed benchmark values reported in Table 4.

**Table 4:** Proposed competency benchmarks for SDSCs and ICC

Metric	Thigh benchmark	Calf benchmark
Interscan area ICC	0.966	0.991
Interscan large-ROI FF ICC	0.994	0.998
Interobserver area ICC	0.903	0.963
Interobserver large-ROI FF ICC	0.988	0.997
Interobserver IM SDSC	0.871	0.923
Interobserver CM SDSC	0.921	0.938

*CM* Combined muscle, *FF* Fat fraction, *ICC* Intraclass correlation coefficient, *IM* Individual muscle, *SDSC* Sørensen-Dice similarity coefficient

### Comparison of reliability of patient and control scans

Reliability metrics differed for control scans compared to against patient scans (Table 5). Control scans showed greater reliability for muscle cross-sectional area measurements with lower interscan standard deviation at thigh and calf level and high interscan ICC at thigh level (Table 5). Interobserver SDSCs were higher for control than patient scans, particularly for thigh muscles (Table 6). When considering large ROI fat fraction measurements, the interscan standard deviation was higher for patients at both thigh and calf level, especially from the small ROI due to inhomogeneity in patients. However, the ICC for fat fraction values were higher in patients, reflecting the greater range of fat fraction values seen in patients.

**Table 5:** Interscan reliability metrics across all observers for control scans *versus* patient scans

	Group	Thigh area	Calf area	Thigh large-ROI FF	Thigh small-ROI FF	Calf large-ROI FF	Calf small-ROI FF
Mean interscan s.d	Control	126 voxels	104 voxels	0.55% ff	0.64% ff	0.42% ff	0.52% ff
	Patient	257 voxels	198 voxels	3.31% ff	10.51% ff	1.63% ff	3.98% ff
Mean ICC	Control	0.992	0.993	0.902	0.690	0.941	0.897
	Patient	0.950	0.994	0.988	0.919	0.999	0.994

*FF* Fat fraction, *ICC* Intraclass correlation coefficient, *ROI* Region of interest, *s.d.* Standard deviation

**Table 6:** Mean and benchmark interobserver individual muscle Sørensen-Dice coefficients (O1−O3 *versus* O5−O8, O10−O11) for control *versus* patient scans

Group	Thigh mean	Thigh benchmark	Calf mean	Calf benchmark
Control	0.941	0.915	0.950	0.939
Patient	0.866	0.816	0.929	0.903

Benchmark is calculated as mean—1.96 × standard deviation

## Discussion

We developed, implemented, and analysed a lower limb muscle MRI segmentation training programme. We established objective criteria to qualitatively and quantitatively identify new observers who were underperforming outliers and propose benchmark reliability coefficients for future inexperienced observers who undertake this training programme.

The interscan reliability observed here for small ROI in the two controls with 11 observers was similar to the larger group of 15 controls we previously reported in whom the interscan standard deviation for fat fraction in controls was 0.58% fat fraction at thigh and 0.64% fat fraction for calf muscles [16]. In the current study, using large ROIs, the reliability was improved a little in controls but markedly in patients (see Tables 5 and 6). This is a potential explanation for the finding that fat fraction derived from large ROI demonstrated superior longitudinal responsiveness than small ROI in our longitudinal Charcot-Marie-Tooth disease/inclusion body myositis study [17].

In addition, some general observations with regards to manual segmentation can be made. First, small ROI are insufficiently reliable both for inter-observer and inter-scan reliability of muscle fat fraction to be considered ideal as an outcome measure in this patient group and should not be used as a primary outcome measure. This is likely due to the inhomogeneity of fat accumulation within patient muscles. Second, calf muscle reliability metrics were higher than those for thigh muscles. This was the case across all reliability metrics. This may be in part due to the lower degree of fat accumulation in calf muscles (20.9% thigh, 13.6% calf) but also probably reflects that calf muscle boundaries are more easily identified than for some thigh muscles (see Fig. 4 for examples). This was reflected in the fact that the combined thigh muscle SDSCs were higher than for individual thigh muscles (*p* < 0.001), whereas for calf muscles the SDSCs were similar (*p* = 0.066). A recent study demonstrated superior longitudinal responsiveness of combined *versus* individual muscle MRI segmentation over 12 months in neuromuscular diseases [21], which may in part be explained by the greater interscan reliability of combined muscle measurements observed in our study. Third, cross-sectional area measurements were less reliable than mean fat fraction measurements, for both interobserver and interscan ICCs (*p* < 0.001 for all). The imaging protocol was not optimised for muscle area as there was a 2-cm distance between slices, and smaller slice gaps or other optimisations should be considered if muscle size parameters are planned as primary outcome measures in clinical trials.

Finally, we have established benchmarks for competent observers in terms of reliability coefficients, including interobserver SDSCs greater than 0.921 for thigh muscles and greater than 0.938 for calf muscles, though this will vary depending on whether pathology is present (Table 6). Even between experienced observers, segmentation overlap is not perfect due to subjective decisions regarding the exact boundary between muscles and between muscle and other tissues, and hence no true perfect standard ROI exists.

However, these data provide a framework for the expected agreement between experienced observers. In addition to assessing future human observers, the same benchmarks could be applied to assess the performance of automated segmentation methods such as using convoluted neural networks [12] resulting in significant time savings which make more complex analysis such as segmentation of three-dimensional images feasible. Furthermore, interobserver reliability metrics are necessarily limited by the ‘expert’ ROI against which they are compared. However, the analysis of test–retest reliability data as in this study, for assessing responsiveness of outcome measures in natural history studies, or treatment efficacy in clinical trials allows the new observer (being human or machine) to outperform the existing methodology by showing improved reliability, responsiveness, or treatment effect.

The major limitation of this study is that these benchmarks are specific to the dataset used in the assessment phase and may not be applicable to other datasets for example subjects with other neuromuscular diseases or sarcopenia. However, the inclusion of two patients with significant intramuscular fat accumulation and two healthy controls mean they are a useful guide to the range of likely interscan and interobserver reliability coefficients expected in other manually segmented data sets. In any quantitative study, the observer should gain experience in imaging of the specific patient population before commencing the definitive segmentations. Future studies should look at interscan and interobserver reliability in other disease groups and also compare with segmentations performed using machine learning algorithms.

In conclusion, this study describes a lower limb muscle MRI segmentation training programme for inexperienced observers. We established objective criteria to identify new observers who were underperforming outliers who require further training and propose benchmark reliability coefficients for future inexperienced observers to successful pass this training programme. The interscan and interobserver reliabilities obtained were very high, particularly with regards muscle fat fraction measurements, which underpins the use of these outcome measures in clinical trials in neuromuscular diseases. The results in this study are necessarily specific to this data set in terms of anatomy, sequences, and pathology. However, the principle of having an adequately trained observer, either human or machine, performing segmentations is clearly a critical part in using quantitative muscle MRI as an outcome measure in neuromuscular diseases, and optimising reproducibility of this step will increase outcome measure responsiveness.

## Data Availability

The datasets used and/or analysed during the current study are available from the corresponding author on reasonable request.

## Abbreviations

ICC: Intraclass correlation coefficient
MRI: Magnetic resonance imaging
O1 − O11: Observers 1 − 11
ROI: Region of interest
SDSC: Sørensen-Dice similarity coefficient
